# *tert*-Butyl 3-oxo-2-oxa-5-aza­bicyclo­[2.2.1]heptane-5-carboxyl­ate

**DOI:** 10.1107/S1600536808030651

**Published:** 2008-09-30

**Authors:** Marie-Charlotte Lechner, Emmanuel Aubert, Gilles Guichard, Claude Didierjean

**Affiliations:** a CNRS, Institut de Biologie Moléculaire et Cellulaire, Laboratoire d’Immunologie et Chimie Thérapeutiques, 15 rue René Descartes, F-67000 Strasbourg, France; b Laboratoire de Cristallographie et Modélisation des Matériaux Minéraux et Biologiques (LCM3B), UMR No. 7036, Nancy Université, Faculté des Sciences et Techniques, BP 239, 54506 Vandoeuvre lès Nancy Cedex, France

## Abstract

The title compound, C_10_H_15_NO_4_, also known as *N*-*tert*-butyl­oxycarbonyl-allohydr­oxy-l-proline lactone, is quite similar to *N*-acetyl-allohydr­oxy-l-proline lactone [Lenstra, Petit & Geise (1979 ▶). *Cryst. Struct. Commun*. **8**, 1023–1029], whereby both carbonyl groups point roughly in the same direction because of the *trans* conformation of the peptide bond.

## Related literature

For general background, see: Allen (2002 ▶). For related structures, see: Didier *et al.* (2004 ▶); Lenstra *et al.* (1979 ▶); Papaioannou *et al.* (1989 ▶). For related synthesis, see: Gómez-Vidal & Silverman (2001 ▶). For related literature, see: Flack & Schwarzenbach (1988 ▶).
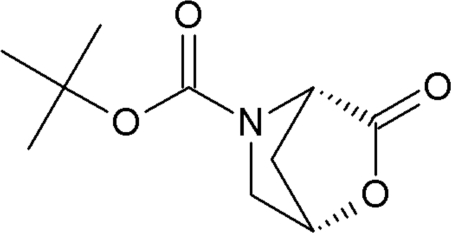


## Experimental

### 

#### Crystal data

C_10_H_15_NO_4_
*M* *_r_* = 213.23
Monoclinic, 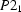

*a* = 6.0710 (7) Å
*b* = 9.3703 (11) Å
*c* = 9.3002 (10) Å
β = 100.013 (5)°
*V* = 521.00 (10) Å^3^
*Z* = 2
Mo *K*α radiation
μ = 0.10 mm^−1^
*T* = 100 (2) K
0.3 × 0.2 × 0.2 mm

#### Data collection

Nonius KappaCCD area-detector diffractometer
Absorption correction: none
5951 measured reflections
1143 independent reflections
1054 reflections with *I* > 2σ(*I*)
*R*_int_ = 0.066

#### Refinement

*R*[*F* ^2^ > 2σ(*F* ^2^)] = 0.052
*wR*(*F* ^2^) = 0.102
*S* = 1.20
1143 reflections
139 parameters
1 restraint
H-atom parameters constrained
Δρ_max_ = 0.23 e Å^−3^
Δρ_min_ = −0.22 e Å^−3^

### 

Data collection: *COLLECT* (Nonius, 1998 ▶); cell refinement: *SCALEPACK* (Otwinowski & Minor, 1997 ▶); data reduction: *DENZO* (Otwinowski & Minor, 1997 ▶) and *SCALEPACK*; program(s) used to solve structure: *SIR92* (Altomare *et al.*, 1994 ▶); program(s) used to refine structure: *SHELXL97* (Sheldrick, 2008 ▶); molecular graphics: *ORTEP-3 for Windows* (Farrugia, 1997 ▶); software used to prepare material for publication: *WinGX* (Farrugia, 1999 ▶).

## Supplementary Material

Crystal structure: contains datablocks global, I. DOI: 10.1107/S1600536808030651/ww2125sup1.cif

Structure factors: contains datablocks I. DOI: 10.1107/S1600536808030651/ww2125Isup2.hkl

Additional supplementary materials:  crystallographic information; 3D view; checkCIF report

## Figures and Tables

**Fig. 1.**
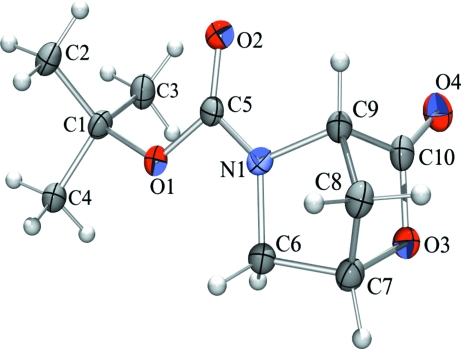
The molecular structure of title compound showing the atom-numbering scheme. All non-H atoms are represented by 50% probability displacement ellipsoids.

## supplementary crystallographic information

### Comment

*N*-*tert*-Butyloxycarbonyl-allohydroxy-*L*-proline lactone is prepared in one step under Mitsunobu conditions starting from corresponding *trans*-4-hydroxyproline. As previously described (Gómez-Vidal & Silverman, 2001), this lactone is a useful derivative that can be readily transformed to *N*-Boc-*cis*-4-hydroxyl-*L*-prolinemethyl ester by quantitative *trans*-esterification with methanol in the presence of sodium azide. Further transformation of the hydroxyl group to an azido group in the presence of diphenylphosphorylazide (DPPA) under Mistunobu conditions affords *N*-Boc-*trans*-4-azido-*L*-proline methyl ester, a useful building block for the preparation of 4-aminoproline containing molecules.

A search of the Cambridge Structural Database (CSD, Version 5.29; Allen, 2002) for allohydroxy-*L*-proline lacton gave rise to 3 hits: (1*S*,4S)-*N*-acetyl-3-oxo-5-aza-2-oxabicyclo[2.2.1]heptane (Lenstra *et al.*, 1979); *N*-triphenylmethyl-2-oxa-5-azabicyclo[2.2.1]heptan-3-one (Papaioannou *et al.*, 1989); *tert*-butyl 7-chloro-6-methyl-2,3-dihydro-2-oxo-6*H*-3,10*b*-methano-1,4- dioxazino[3,2-*c*](2,1)benzoxazine-4(4*aH*)-carboxylate (Didier *et al.*, 2004). In the four structures the pyrrolidine ring (N1/C6/C7/C8/C9 numbering in the title compound) adopts the same envelope conformation with C8 out of the mean plane defined by N1, C6, C7 and C9. Three structures consists of an amide bond: the title compound, the *N*-acetyl-allohydroxy-*L*-proline lacton and the *tert*-butyl- 7-chloro-6-methyl-2,3-dihydro-2-oxo-6*H*-3,10*b*-methano-1,4- dioxazino(3,2-*c*)(2,1)benzoxazine-4(4*aH*)-carboxylate. The two first structures exhibit a quite similar structure with a nearly planar *trans-* amide bond. In the last one, the peptide bond is *cis-* and the nitrogen atom of the pyrrolidine ring exhibits an observable pyramidalization. Indeed, the sum of bond angles around the nitrogen atom is of 347.9° whereas of 357.9° and 357.4° in the two first structures.

### Experimental

The title compound was prepared in 80% from *N*-Boc-*trans*-4-hydroxyproline following the a described procedure (Gómez-Vidal & Silverman, 2001) and was crystallized by slow evaporation of a cyclohexane/ethyl acetate (3:2, *v*/*v*) solution.

### Refinement

Because of the lack of any significant anomalous dispersion effects, the absolute configurations of the title compound could not be determined from the diffraction experiments but was known from the method of synthesis. The origin was fixed by floating-origin restraints (Flack & Schwarzenbach, 1988). All H atoms were located in difference Fourier maps. The C-bonded H atoms were placed at calculated positions and refined using a riding model, with C—H distances of 0.93–0.96 Å. The H-atom *U*_iso_ parameters were fixed at 1.2U_eq_(C) for methine and methylene C—H and at 1.5U_eq_(C) for methyl C—H.

### Crystal data

**Table:**

C_10_H_15_NO_4_	*F*(000) = 228
*M**_r_* = 213.23	*D*_x_ = 1.359 Mg m^−^^3^
Monoclinic, *P*2_1_	Mo *K*α radiation, λ = 0.7107 Å
Hall symbol: P 2yb	Cell parameters from 11845 reflections
*a* = 6.0710 (7) Å	θ = 0.4–26.4°
*b* = 9.3703 (11) Å	µ = 0.11 mm^−^^1^
*c* = 9.3002 (10) Å	*T* = 100 K
β = 100.013 (5)°	Prism, colourless
*V* = 521.00 (10) Å^3^	0.3 × 0.2 × 0.2 mm
*Z* = 2	

### Data collection

**Table:**

Nonius KappaCCD area-detector diffractometer	1054 reflections with *I* > 2σ(*I*)
Radiation source: fine-focus sealed tube	*R*_int_ = 0.066
graphite	θ_max_ = 26.6°, θ_min_ = 3.1°
ω and φ scans	*h* = −7→7
5951 measured reflections	*k* = −11→11
1143 independent reflections	*l* = −11→11

### Refinement

**Table:**

Refinement on *F*^2^	1 restraint
Least-squares matrix: full	H-atom parameters constrained
*R*[*F*^2^ > 2σ(*F*^2^)] = 0.052	*w* = 1/[σ^2^(*F*_o_^2^) + (0.0035*P*)^2^ + 0.6362*P*] where *P* = (*F*_o_^2^ + 2*F*_c_^2^)/3
*wR*(*F*^2^) = 0.102	(Δ/σ)_max_ < 0.001
*S* = 1.20	Δρ_max_ = 0.23 e Å^−^^3^
1143 reflections	Δρ_min_ = −0.22 e Å^−^^3^
139 parameters	

### Special details

**Table:**

Geometry. All e.s.d.'s (except the e.s.d. in the dihedral angle between two l.s. planes) are estimated using the full covariance matrix. The cell e.s.d.'s are taken into account individually in the estimation of e.s.d.'s in distances, angles and torsion angles; correlations between e.s.d.'s in cell parameters are only used when they are defined by crystal symmetry. An approximate (isotropic) treatment of cell e.s.d.'s is used for estimating e.s.d.'s involving l.s. planes.

### Fractional atomic coordinates and isotropic or equivalent isotropic displacement parameters (Å^2^)

**Table:**

	*x*	*y*	*z*	*U*_iso_*/*U*_eq_	
O1	0.3991 (4)	0.3322 (3)	0.3133 (3)	0.0243 (6)	
O2	0.0303 (4)	0.2997 (3)	0.2103 (3)	0.0280 (7)	
O3	0.3864 (4)	−0.0967 (3)	0.1079 (3)	0.0262 (7)	
O4	0.0299 (5)	−0.1077 (3)	0.1467 (3)	0.0319 (7)	
N1	0.3137 (5)	0.1896 (4)	0.1226 (4)	0.0230 (7)	
C1	0.3539 (7)	0.4316 (4)	0.4288 (4)	0.0249 (9)	
C2	0.2354 (7)	0.5650 (4)	0.3607 (5)	0.0306 (10)	
H2A	0.3194	0.6052	0.2893	0.046*	
H2B	0.2265	0.6356	0.4372	0.046*	
H2C	0.0842	0.54	0.3117	0.046*	
C3	0.2204 (8)	0.3563 (5)	0.5297 (5)	0.0315 (10)	
H3A	0.075	0.3274	0.4744	0.047*	
H3B	0.1977	0.4212	0.6085	0.047*	
H3C	0.3024	0.2717	0.5715	0.047*	
C4	0.5884 (6)	0.4670 (4)	0.5062 (5)	0.0266 (9)	
H4A	0.6574	0.3816	0.5556	0.04*	
H4B	0.5813	0.5423	0.5784	0.04*	
H4C	0.678	0.5001	0.4347	0.04*	
C5	0.2283 (6)	0.2759 (4)	0.2159 (4)	0.0227 (8)	
C6	0.5472 (7)	0.1381 (4)	0.1373 (5)	0.0250 (9)	
H6A	0.6091	0.1101	0.239	0.03*	
H6B	0.646	0.21	0.1032	0.03*	
C7	0.5098 (7)	0.0093 (4)	0.0356 (4)	0.0265 (9)	
H7	0.648	−0.0281	0.004	0.032*	
C8	0.3299 (7)	0.0625 (5)	−0.0887 (4)	0.0259 (9)	
H8A	0.3762	0.1478	−0.1388	0.031*	
H8B	0.273	−0.0127	−0.1603	0.031*	
C9	0.1701 (6)	0.0969 (4)	0.0167 (4)	0.0233 (9)	
H9	0.0204	0.1361	−0.0275	0.028*	
C10	0.1710 (7)	−0.0459 (4)	0.0960 (4)	0.0256 (9)	

### Atomic displacement parameters (Å^2^)

**Table:**

	*U*^11^	*U*^22^	*U*^33^	*U*^12^	*U*^13^	*U*^23^
O1	0.0249 (14)	0.0180 (14)	0.0299 (14)	−0.0012 (12)	0.0044 (12)	−0.0053 (12)
O2	0.0234 (14)	0.0253 (16)	0.0352 (16)	0.0018 (13)	0.0050 (12)	−0.0054 (13)
O3	0.0253 (15)	0.0189 (14)	0.0349 (16)	−0.0018 (13)	0.0067 (12)	0.0007 (13)
O4	0.0344 (17)	0.0263 (16)	0.0359 (16)	−0.0089 (14)	0.0091 (13)	0.0022 (14)
N1	0.0215 (17)	0.0192 (17)	0.0275 (18)	0.0024 (14)	0.0017 (14)	−0.0023 (15)
C1	0.027 (2)	0.018 (2)	0.031 (2)	0.0018 (17)	0.0092 (17)	−0.0050 (17)
C2	0.034 (2)	0.018 (2)	0.039 (2)	0.0029 (18)	0.0049 (19)	−0.0017 (19)
C3	0.037 (2)	0.023 (2)	0.035 (2)	−0.0045 (18)	0.0101 (19)	−0.0047 (19)
C4	0.026 (2)	0.020 (2)	0.034 (2)	−0.0009 (17)	0.0064 (17)	−0.0039 (18)
C5	0.023 (2)	0.0157 (19)	0.029 (2)	−0.0012 (16)	0.0028 (16)	0.0015 (16)
C6	0.024 (2)	0.021 (2)	0.030 (2)	−0.0009 (17)	0.0057 (16)	−0.0024 (17)
C7	0.028 (2)	0.022 (2)	0.030 (2)	−0.0006 (18)	0.0099 (19)	−0.0045 (18)
C8	0.031 (2)	0.019 (2)	0.028 (2)	−0.0005 (17)	0.0056 (17)	−0.0007 (18)
C9	0.0238 (19)	0.0187 (19)	0.027 (2)	−0.0022 (17)	0.0049 (17)	−0.0039 (16)
C10	0.031 (2)	0.020 (2)	0.025 (2)	−0.0038 (18)	0.0024 (17)	−0.0047 (17)

### Geometric parameters (Å, °)

**Table:**

O1—C5	1.359 (5)	C3—H3B	0.98
O1—C1	1.484 (5)	C3—H3C	0.98
O2—C5	1.215 (5)	C4—H4A	0.98
O3—C10	1.377 (5)	C4—H4B	0.98
O3—C7	1.475 (5)	C4—H4C	0.98
O4—C10	1.196 (5)	C6—C7	1.526 (6)
N1—C5	1.353 (5)	C6—H6A	0.99
N1—C9	1.478 (5)	C6—H6B	0.99
N1—C6	1.481 (5)	C7—C8	1.529 (6)
C1—C4	1.516 (5)	C7—H7	1
C1—C3	1.516 (6)	C8—C9	1.528 (6)
C1—C2	1.524 (6)	C8—H8A	0.99
C2—H2A	0.98	C8—H8B	0.99
C2—H2B	0.98	C9—C10	1.528 (6)
C2—H2C	0.98	C9—H9	1
C3—H3A	0.98		
			
C5—O1—C1	120.7 (3)	O2—C5—O1	126.3 (4)
C10—O3—C7	106.3 (3)	N1—C5—O1	109.0 (3)
C5—N1—C9	122.1 (3)	N1—C6—C7	99.4 (3)
C5—N1—C6	127.1 (3)	N1—C6—H6A	111.9
C9—N1—C6	108.3 (3)	C7—C6—H6A	111.9
O1—C1—C4	101.8 (3)	N1—C6—H6B	111.9
O1—C1—C3	110.0 (3)	C7—C6—H6B	111.9
C4—C1—C3	111.5 (4)	H6A—C6—H6B	109.6
O1—C1—C2	110.3 (3)	O3—C7—C6	106.4 (3)
C4—C1—C2	110.7 (3)	O3—C7—C8	102.2 (3)
C3—C1—C2	112.0 (3)	C6—C7—C8	102.7 (3)
C1—C2—H2A	109.5	O3—C7—H7	114.7
C1—C2—H2B	109.5	C6—C7—H7	114.7
H2A—C2—H2B	109.5	C8—C7—H7	114.7
C1—C2—H2C	109.5	C9—C8—C7	91.9 (3)
H2A—C2—H2C	109.5	C9—C8—H8A	113.3
H2B—C2—H2C	109.5	C7—C8—H8A	113.3
C1—C3—H3A	109.5	C9—C8—H8B	113.3
C1—C3—H3B	109.5	C7—C8—H8B	113.3
H3A—C3—H3B	109.5	H8A—C8—H8B	110.6
C1—C3—H3C	109.5	N1—C9—C10	103.9 (3)
H3A—C3—H3C	109.5	N1—C9—C8	100.6 (3)
H3B—C3—H3C	109.5	C10—C9—C8	100.1 (3)
C1—C4—H4A	109.5	N1—C9—H9	116.5
C1—C4—H4B	109.5	C10—C9—H9	116.5
H4A—C4—H4B	109.5	C8—C9—H9	116.5
C1—C4—H4C	109.5	O4—C10—O3	122.4 (4)
H4A—C4—H4C	109.5	O4—C10—C9	132.2 (4)
H4B—C4—H4C	109.5	O3—C10—C9	105.4 (3)
O2—C5—N1	124.7 (4)		
			
C5—O1—C1—C4	−178.9 (3)	O3—C7—C8—C9	53.1 (3)
C5—O1—C1—C3	62.7 (5)	C6—C7—C8—C9	−57.1 (3)
C5—O1—C1—C2	−61.3 (4)	C5—N1—C9—C10	−93.9 (4)
C9—N1—C5—O2	−9.6 (6)	C6—N1—C9—C10	69.1 (4)
C6—N1—C5—O2	−169.3 (4)	C5—N1—C9—C8	162.7 (3)
C9—N1—C5—O1	171.3 (3)	C6—N1—C9—C8	−34.2 (4)
C6—N1—C5—O1	11.6 (5)	C7—C8—C9—N1	53.9 (3)
C1—O1—C5—O2	0.9 (6)	C7—C8—C9—C10	−52.5 (3)
C1—O1—C5—N1	179.9 (3)	C7—O3—C10—O4	−178.8 (4)
C5—N1—C6—C7	159.9 (4)	C7—O3—C10—C9	−1.3 (4)
C9—N1—C6—C7	−2.1 (4)	N1—C9—C10—O4	109.5 (5)
C10—O3—C7—C6	73.2 (4)	C8—C9—C10—O4	−146.8 (4)
C10—O3—C7—C8	−34.2 (4)	N1—C9—C10—O3	−67.7 (3)
N1—C6—C7—O3	−69.0 (4)	C8—C9—C10—O3	36.1 (4)
N1—C6—C7—C8	38.0 (4)		
